# Sol-gel synthesis of thorn-like ZnO nanoparticles endorsing mechanical stirring effect and their antimicrobial activities: Potential role as nano-antibiotics

**DOI:** 10.1038/srep27689

**Published:** 2016-06-28

**Authors:** Mohd Farhan Khan, Akhter H. Ansari, M. Hameedullah, Ejaz Ahmad, Fohad Mabood Husain, Qamar Zia, Umair Baig, Mohd Rehan Zaheer, Mohammad Mezbaul Alam, Abu Mustafa Khan, Zeid A. AlOthman, Iqbal Ahmad, Ghulam Md Ashraf, Gjumrakch Aliev

**Affiliations:** 1 Nano Solver Lab, Department of Mechanical Engineering, Z. H. College of Engineering & Technology, Aligarh Muslim University, Aligarh-202002, India; 2 Faculty of Science, Gagan College of Management & Technology (GCMT), Aligarh-202002, India; 3 Department of Biological Sciences, University of Toledo, Toledo, OH43606, USA; 4 Department of Agricultural Microbiology, Aligarh Muslim University, Aligarh-202002, India; 5 Department of Food Science and Nutrition, College of Food and Agriculture, King Saud University, Riyadh-11451, Kingdom of Saudi Arabia; 6 Interdisciplinary Biotechnology Unit, Aligarh Muslim University, Aligarh-202002, India; 7 Department of Biotechnology, Gagan College of Management and Technology (GCMT), Aligarh, India; 8 Center of Excellence for Scientific Research Collaboration with MIT, King Fahd University of Petroleum and Minerals, Dhahran 31261, Saudi Arabia; 9 Advanced Materials Research Chair, Chemistry Department, College of Sciences, Building 5, King Saud University, Riyadh-11451, Kingdom of Saudi Arabia; 10 Department of Chemistry, Aligarh Muslim University, Aligarh-202002, India; 11 King Fahd Medical Research Center, King Abdulaziz University, Jeddah, Saudi Arabia; 12 GALLY International Biomedical Research Consulting LLC., 7733 Louis Pasteur Drive, #330, San Antonio, TX, 78229, USA; 13 School of Health Science and Healthcare Administration, University of Atlanta, E. Johns Crossing, #175, Johns Creek, GA, 30097, USA; 14 Institute of Physiologically Active Compounds, Russian Academy of Sciences, Chernogolovka, 142432, Russia

## Abstract

The effect of mechanical stirring on sol-gel synthesis of thorn-like ZnO nanoparticles (ZnO-NPs) and antimicrobial activities is successfully reported in this study. The in-house synthesized nanoparticles were characterized by XRD, SEM, TEM, FTIR, TGA, DSC and UV-visible spectroscopy. The X-Ray Diffraction analysis revealed the wurtzite crystal lattice for ZnO-NPs with no impurities present. The diametric measurements of the synthesized thorn-like ZnO-NPs (morphology assessed by SEM) were well accounted to be less than 50 nm with the help of TEM. Relative decrease in aspect ratio was observed on increasing the agitation speed. The UV-visible spectroscopy showed the absorption peaks of the ZnO-NPs existed in both UVA and UVB region. A hypsochromic shift in λmax was observed when stirring pace was increased from 500 rpm to 2000 rpm. The FTIR spectroscopy showed the absorption bands of the stretching modes of Zn-O between 500 cm^−1^ to 525 cm^−1^. The Thermal analysis studies revealed better stability for ZnO-NPs prepared at 2000 rpm (ZnO-2000 rpm). TGA revealed the weight loss between two main temperatures ranges viz. around (90 °C–120 °C) and (240 °C–280 °C). Finally, the effect of ZnO-NPs prepared at different stirring conditions on the growth of Gram-positive (Bacillus subtilis), Gram-negative (Escherichia coli) bacteria and a fungi (Candida albicans) were examined; which showed good antibacterial as well as antifungal properties. These findings introduce a simple, inexpensive process to synthesize ZnO-NPs using conventional methods without the use of sophisticated equipments and its application as a potent nano-antibiotic.

In the past decade, several research groups have developed metal oxide nanoparticles using savvy routes^1^,^2^. Among them, a significant category of zinc oxide nanoparticles (ZnO-NPs) have gained importance since few years. Food and Drug Administration (FDA, USA) has categorized zinc oxide (ZnO) as “generally recognized as safe” (GRAS) (21CFR182.8991). ZnO generates blue-green luminescence while absorbing in the ultraviolet (UV) region. This property is exploited for its use in sunscreens^3^, textile industries^4^, catalysts^5^, sensors^6^ and photodetectors^7^. The presence of vacant and uncoordinated atoms at edges as well as large surface area to volume ratio arising due to nanoparticulate organization augments the action of ZnO. Several reports have associated the effective antimicrobial activity of ZnO with its chemical and physical properties^8^. ZnO-NPs exhibit activity against broad spectrum of microorganisms^9^,^10^,^11^,^12^, and have been widely used as active constituent for topical lotions, ointments and creams^13^,^14^. ZnO-NPs aqueous suspension displayed better antibacterial efficacy than its TiO_2_ counterpart^15^. A plethora of techniques including sol-gel method^16^, chemical vapor deposition^17^, pulsed laser deposition^18^, sputtering^19^, hydrothermal synthesis^20^, and oxidation of metallic zinc powder^21^ have been exploited to prepare ZnO in diverse appearances and sizes for various applications.

A simple, well-controlled conventional synthesis process at near-room temperature can be utilized for cost-effective production of ZnO-NPs and its use in functions of biological relevance. In this study, the fabrication of thorn-like ZnO-NPs was done by sol-gel method while varying the stirring conditions (viz. 500 rpm, 1000 rpm, 1500 rpm and 2000 rpm). The starting reagents used for this synthesis were Zinc acetate dihydrate (ZAD), and NaOH while cetyltrimethyammonium bromide (CTAB) was used as a capping agent. The in-house prepared nanoparticles were characterized by X-ray diffraction (XRD) analysis, scanning electron microscopy (SEM), transmission electron microscopy (TEM), Fourier transform infrared spectroscopy (FTIR), thermo-gravimetric analysis (TGA), differential thermal analysis (DTA), differential scanning calorimetric (DSC) and UV-visible spectroscopy. Furthermore, the antimicrobial activity of the thorn-like ZnO-NPs, was assessed against both Gram-positive & Gram-negative bacteria (*Bacillus subtilis* and *Escherichia coli* respectively) and fungi (*Candida albicans*).

## Materials and Methods

### Materials

Zinc acetate dihydrate [Zn(CH_3_COO)_2_·2H_2_O] and sodium hydroxide (NaOH) were purchased from Merck (India), cetyltrimethylammonium bromide (CTAB) was from Loba (India) while nutrient agar (NA), nutrient broth (NB), peptone yeast extract agar, sabouraud dextrose agar (SDA) and agar was from HiMedia (India). The chemicals were of analytical grade and were utilized without additional purification. The glasswares were properly washed, sanitized and autoclaved.

### Synthesis of thorn-like ZnO nanoparticles

To prepare thorn-like ZnO-NPs, 0.2 g of CTAB was added to 100 mL of bi-distilled (BD) water, in a flat-bottomed flask. To this, 10 mL of 1% Zn(CH_3_COO)_2_·2H_2_O solution was added. The whole apparatus was kept on mechanical stirrer under different stirring conditions viz. 500 rpm, 1000 rpm, 1500 rpm and 2000 rpm. Subsequently, the NaOH solution was added drop-wise to the cocktail. Within few minutes of agitation, the colloidal solution turned milky. The setup was retained as such for half an hour. The system was then cooled, centrifuged, dried at room temperature in a desiccator, and washed with BD water and methanol. No complex treatment (for example, high temperature and high pressure, use of inert gas) was utilized for the synthesis. Finally, the samples of ZnO-NPs were stored in amber colour container until further use.

### X-Ray Diffraction Studies

The structural characterization of the ZnO-NPs was performed by X-ray diffraction (XRD) and recorded as a function of 2θ angle. All the diffraction patterns were prepared as step-scans. XRD was performed in the 2θ range of 30°–70° with a step size of 0.01° and a scanning rate of 0.02 steps/second using a monochromatized X-ray beam with nickel-filtered Cu-K_α_ (λ = 1.54178 Å) radiation by an X-ray diffractometer (D8 ADVANCE; Bruker AXS Inc, Madison, WI, USA) operating at a voltage of 40 kV and a current of 40 kA. The data were smoothed with a weighted moving average to obtain a diffractogram.

The ZnO-NPs crystallite size (D) was calculated from the highest intense peak (101) using the Debye–Scherrer equation:


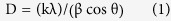


where *k* is the proportionality constant (*k* = 0.9); *λ* is the X-ray wavelength coming from Cu-K_α_; *β* is the full width at half maxima (FWHM) of the diffraction peak in radians; *θ* is the Braggs’ angle in degrees^22^. The calculation was done using the software, DIFFRACplus (Bruker AXS Inc).

### Electron Microscopy

The size characterization of ZnO-NPs was performed using a Transmission electron microscope (TEM) (JEM-2100F; Jeol, Tokyo, Japan)^23^ featuring ultra high resolution and rapid data acquisition. The lyophilized ZnO-NPs was suspended in 20 mM phosphate-buffered saline (PBS, pH 7.4) and a drop of the nanoparticles was mounted on a clear glass stub, air-dried, and coated with gold-palladium alloy using a sputter coater. An accelerating voltage of 200 kV was used for imaging.

The surface morphology of ZnO-NPs was investigated with the help of Scanning electron microscope (SEM) (JSM-6510 LV, Jeol, Tokyo, Japan)^16^. The samples were examined in the microscope with an acceleration voltage of 5 kV and a current of 10 μA. The samples were made more viable by coating with gold sputter.

### Dynamic light scattering measurements.

 DLS was performed using DynaPro-TC-04 system (Protein Solutions, Wyatt technology, Santa Barbara, CA, USA) equipped with a temperature-controlled microsampler.

### UV-visible spectroscopy

UV–vis absorption spectra was measured using Lambda 850 UV/vis spectrophotometer (Perkin-Elmer Life and Analytical Sciences, CT, USA) in the range of 300–600 nm operating at a resolution of 1 nm, in the quartz cuvette of 1 cm path length. The BD water was used as a reference material for background correction.

### FT-IR spectroscopy

The Fourier transform infrared (FT-IR) spectra was taken with the help of Nicolet iS 10 spectrometer (Thermo Scientific, Massachusetts, USA). The FT-IR spectra of ZnO-NPs were recorded in spectroscopic grade KBr pellet (used at the ratio of 1:100) at a resolution of 4 cm^−1^ in the frequency range from 400 to 4000 cm^−1^ in diffuse reflectance mode. The identification of various functional groups and chemical structures in the ZnO-NPs was done by analyzing absorption of electromagnetic waves at distinctive frequencies & intensities. The average of three scans for each sample was taken for the peak identification.

### Thermal behavior of ZnO nanoparticles

#### Thermogravimetric analysis (TGA) and differential thermal analysis (DTA)

The degradation process and the thermal stability of ZnO-NPs were investigated by TGA and DTA using thermogravimetric analyzer (SDT Q-600 TA Instruments, Artisan Technology Group, IL, USA). Initial sample weight was set as 5–8 mg for each operation. The sample was heated from ~24 °C to ~900 °C at the rate of 10 °C/min in the nitrogen atmosphere at the flow rate of 200 ml/min.

#### Differential scanning calorimetric (DSC) analysis

DSC measurements of ZnO-NPs were recorded on a scanning calorimeter Q-100 (TA instruments, DE, USA). The energy changes associated with transitions were recorded at the temperature range of 20 to 600 °C. Samples of known weight encapsulated in standard aluminium pans were placed in the sample holder and analyzed^24^.

#### Antimicrobial activity of thorn-like ZnO nanoparticles

The thorn-like ZnO-NPs synthesized at different stirring conditions (viz. 500 rpm, 1000 rpm, 1500 rpm and 2000 rpm) were tested for bactericidal and fungicidal activity by disc-diffusion method^25^ as well as growth inhibition studies against both Gram-positive (*Bacillus subtilis*) and Gram-negative (*Escherichia coli*) bacteria as well as fungi (*Candida albicans*) as standardized in our lab^16^. In paper disc diffusion, diluted bacterial culture (0.1 mL) was plated uniformly with the help of a spreader on nutrient agar (NA) plates. Sterile 8 mm discs (HiMedia Laboratories Pvt Ltd, Mumbai, India) infused with the ZnO-NPs were placed on the plates, which were then incubated at 37 °C for 24 hours. For antifungal activity of ZnO-NPs, the fungal culture were spread on sabouraud dextrose agar (SDA) plates and incubated for 18–48 hours. An antibiotic disc (0.03 mg/disc; HiMedia Laboratories Pvt Ltd), doxycycline (for bacteria), and nystatin (for fungi) were used as standard. The diameters of the resulting inhibition zones (in mm) of microbial growth were measured for the determination of antibacterial and antifungal activities.

For growth inhibition kinetics, the bacteria were first grown on solid nutrient agar medium. Then, fresh colonies were picked from the agar plates and inoculated into 100 ml of nutrient broth (NB) medium. Growth was monitored at every 2 h under UV-visible spectrophotometer (Electronics Corporation of India Limited, UV-2550) till 26 h. Similarly, antifungal tests were performed by measuring the growth curve of *C. albicans* incubated in Yeast, peptone, dextrose (YPD) medium containing different ZnO-NPs and growth was read every 2 h. The experiments were repeated thrice and results are expressed as mean ± SD.

CLSI (formerly NCCLS) broth dilution method^26^,^27^ was utilized for determining the MIC values against *Candida* and bacterial pathogens. MIC was defined as the minimum concentration of ZnO-NPs at which there was no visible growth of the test pathogens^28^. Details of these procedures have been provided in the supplementary section.

#### Statistical analysis

All the results are reported as Mean ± standard deviation (SD) of at least three replicates. Data were subjected to one-way analysis of variance (ANOVA) and the least significant difference (LSD).

## Results and Discussion

ZnO nanoparticles (ZnO-NPs) have been synthesized in various forms and structures such as nanoflowers, nanorods, nanowhiskers, nanobelts, nanotubes, nanorings, nanocolumns, etc^29^,^30^,^31^,^32^,^33^,^34^. They have been prepared employing diverse techniques including sol-gel method^16^, solution precipitation method^35^, spray pyrolysis^36^, hydrothermal method^37^, microwave assisted technique^38^ and many more. The processes generally used for ZnO-NPs preparation employ change in various reaction parameters, such as change in temperatures ranging from low to ~1,500 °C, change in pressures from 1 atm to a few mTorr and so on; with majority of them requiring complicated and expensive setups for carrying out the reaction. Incidentally, several reports on production of flower-like ZnO nanorods hydrothermally at <120 °C and a pH of 13.5^39^; sodium dodecyl sulfate derived synthesis of chrysanthemum-like ZnO nanorods at 120 °C for 24 hours^40^ and 10 hours^41^, and the creation of flower-like ZnO particles via sonochemical method for^42^, are neither realistic nor cost-effective and present a few cases of complicated syntheses process. Therefore, there is a paramount need for a cheaper and more user-friendly synthesis system operable at low temperature, while considering the size and morphology of the crystal, and pH of the solution, in order to avoid pricey and complex preparatory routes.

We have previously reported the synthesis of ZnO-NPs at low temperatures, while avoiding sophisticated equipments^16^. The low temperature solution synthesis has been used by researchers to produce flower-shaped ZnO nanostructures using the zinc acetate dihydrate and NaOH^43^. Aqueous precipitation methodology at low temperatures proves to be versatile, economical route and bestows a high yield^44^; resulting in the surface of nanoparticles being coated with high concentration of zinc hydroxide^44^,^45^,^46^. Lately, our group has also developed flower-shaped ZnO nanoparticles at different temperatures (at 25 °C, 35 °C, 55 °C, and 75 °C) utilizing simple sol–gel synthesis process. It was found that nanoparticles synthesized at near-room temperatures exhibited greater activity against microbes^16^. Several studies have employed the use of stirring during preparation of ZnO-NPs^47^,^48^,^49^. However, the method of producing ZnO-NPs exploiting stirring effect as the prime parameter has not been reported till now. Herein, the effect of different stirring conditions on the size, morphology, thermal stability and antimicrobial activity of ZnO-NPs has been evaluated.

### X-ray diffraction analysis

**Figure 1:**
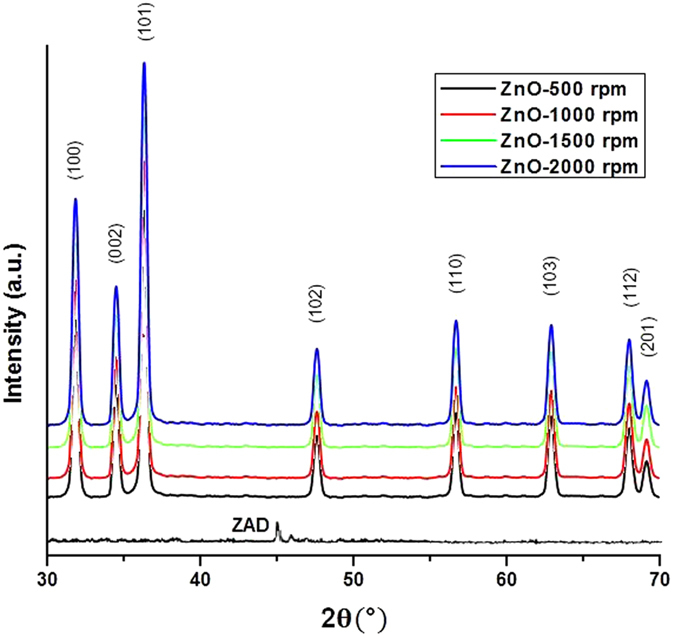
XRD of ZnO nanoparticles prepared at different stirring conditions.

A detailed investigation was performed to characterize the ZnO-NPs synthesized at varying stirring conditions (viz. 500 rpm, 1000 rpm, 1500 rpm and 2000 rpm). The phase and crystal parameters of the synthesized sample were explored by X-ray diffraction patterns (Fig. 1). A most intense peak at 2θ = 36.2° is obtained along (101) orientation. The sharpness and strong intensity of ZnO diffraction peaks indicate that the synthesized ZnO sample is well crystallized. The peaks obtained for ZnO-NPs at 2θ = 31.9°. 34.7°, 36.2°, 47.6°, 56.6°, 63.1°, 67.9° and 69.0° matches up with the lattice planes (100), (002), (101), (102), (110), (103), (112) and (201) respectively. This is in accordance with several previous reports^50^,^51^. By applying the Debye–Scherrer equation^22^, the average particle size were found to be ~25 nm, ~20 nm, ~7 nm and ~3 nm, respectively at 500, 1000, 1500 and 2000 rpm from the FWHM of dominant peak (101). Therefore, the peak widths are inversely related to the crystal sizes^52^.

The (hkl) values agreed well with the standard card of ZnO powder sample (The International Centre for Diffraction Data, PA, USA; ICDD card No: 080-0075); with the reflections assigned to the powder pattern for the pure hexagonal wurtzite having lattice constant (c/a) of 1.60. Additionally, the peaks obtained at planes (100), (002), and (101) are also suggestive of pure wurtzite structure of ZnO^52^,^53^. No additional peaks related to impurities were noticed, validating the pure form of the ZnO-NPs. The study also revealed the broadening of peaks on moving from sample ZnO-500 rpm towards sample ZnO-2000 rpm; hence signifying a drop in the thickness of sample structures with the concurrent increase in sharpness of thorn like morphology of ZnO-NPs.

ZnO exists in three crystal lattice structures i.e. wurtzite (B4), zinc-blende (B3) and rock-salt (B1)^54^,^55^. The ZnO zinc blende form can only be made stable by growing ZnO on cubic lattice structure; the rock salt or Rochelle salt (NaCl) structure is metastable phase obtained only at high-pressure. The wurtzite structure has a hexagonal unit cell with two lattice parameters ‘a’ and ‘c’ in the ratio of c/a = √8/3 (1:633, an ideal value for wurtzite hexagonal structure) and belongs to the space group C^4^_6v_ in the Schoenflies notation and P6_3_mc in the Hermann–Mauguin notation. Each tetrahedral Zn atom is surrounded by four oxygen atoms and vice versa^56^. This structure is thermodynamically stable in an ambient environment^55^, and is represented as a number of alternating planes of Zn and O ions stacked alongside the c-axis.

### Electron microscopy

**Figure 2:**
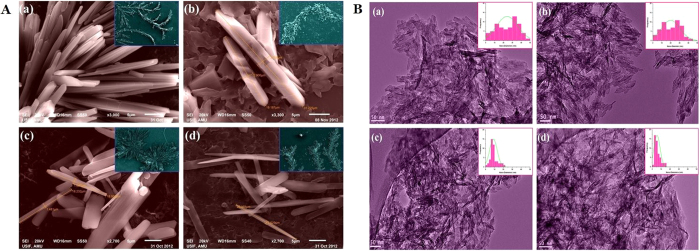
(**A**) SEM images of ZnO nanoparticles prepared at different stirring conditions (a) 500 rpm, (b) 1000 rpm, (c) 1500 rpm and (d) 2000 rpm. The large colonies of the respective samples are shown in insets. (**B)** TEM images of ZnO nanoparticles prepared at different stirring conditions (a) 500 rpm, (b) 1000 rpm, (**c**) 1500 rpm and (d) 2000 rpm. Insets show the particle-size distribution curves.

SEM characterization results showed the appearance of thorn like morphology for ZnO-NPs and the desired structures appeared to be smaller and sharper for the sample prepared at higher stirring rpm (Fig. 2A). Here, the SEM patterns agree well with the XRD results. The TEM images (embedded with particle size distribution curves in inset) identified the diametric evidences between 3–50 nm for the various samples prepared under different stirring conditions (Fig. 2B). The thickness of the samples was found to be inversely proportional to the speed of stirring (Table 1). The average aspect ratios (L/D; length by diameter) were ~8.6 nm, ~9 nm, ~13 nm and ~18 nm, for ZnO-NPs prepared at 500, 1000, 1500 and 2000 rpm respectively. An increase in the aspect ratio was observed with the increase in speed of agitation; showing an inverse relationship with the crystallite size (Table 1). High stirring condition facilitates proper mixing and better dispersion with the surfactant, leading to the growth along the length (c-axis) while decreasing the diameter. This could explain the concomitant enhancement in aspect ratio on increasing the stirring rate while decreasing the nano-diameters of the particles.

### Spectroscopic studies of ZnO nanoparticles

**Figure 3:**
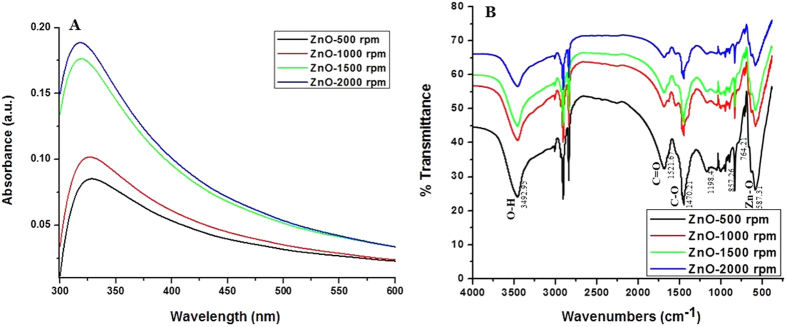
(**A**) UV-vis spectra of ZnO nanoparticles prepared at different stirring conditions. (**B)** FTIR spectra of ZnO nanoparticles prepared at different stirring conditions.

UV-Vis absorption spectrum of ZnO-NPs was taken after few minutes of sample preparation (Fig. 3A). The spectra recorded under different agitation conditions presented noticeable differences in absorbance intensity as well as in wavelength maxima (λ_max_). The main absorption peaks were found to be located around 315–340 nm; peaks of sample ZnO-500 rpm & ZnO-1000 rpm existed in the UV-B region i.e. 320–400 nm and that of sample ZnO-1500 rpm & ZnO-2000 rpm were found in UV-A region i.e. 290–320 nm. A shift in the absorption peaks from UV-B to UV-A region was observed, when one goes from low stirring conditions to high stirring speeds. Moreover, the intensity of absorption peaks was found to be much elevated at 2000 rpm that that at 500 rpm.

At stirring speed of 500 rpm, the ZnO-NPs formed are more agglomerated and thickened with less aspect ratio (L/D fractions), hence absorb at higher wavelength. However, on increasing the stirring speed, the thorn shaped ZnO-NPs are formed more readily (as confirmed by the electron microscopy), with lower agglomeration of particles. This increases the aspect ratios with the proportionate decrease in the diameter; resulting in the shift of λ_max_ from 328 nm to 316 nm, with concomitant increase in the absorbance. The blue shift denotes the decrease in size of particle (as indicated by TEM) and increase in band gap energy^57^. Our findings are in accordance with those from previously reported studies indicating that shifts in the peak is dependent on the size of the crystal, the solvent used, the reaction temperature, the technique of synthesis, and the maturation time of samples^58^,^59^. Moreover, the spectrum of the ZnO-NPs prepared at 500 rpm absorb poorly, while samples synthesized at 2000 rpm exhibited maximum absorption.

The FTIR results were taken at room temperature within the range of 400 cm^−1 ^to 4000 cm^−1^ for thorn-like ZnO samples (Fig. 3B) with different stirring specifications. Metal oxides generally exhibit absorption bands in fingerprint region (i.e. below 1000 cm^−1^), which arise from inter-atomic vibrations. It was found that the peculiar Zinc oxide absorption band with stretching mode of Zn-O was between 500 cm^−1 ^to 590 cm^−1^, which corresponds to E_2_ form of hexagonal ZnO wurtzite crystal (lattice) structure^60^. Peak near 1521.67 and 1470.21 observed in sample corresponds to the asymmetric and symmetric stretching vibrations of C=O group, which may be due to zinc acetate used in reaction^61^. The fundamental mode of vibration near 3492.95 cm^−1^ corresponds to the asymmetric and symmetric stretching H-O-H vibration. In literature, two different OH group interactions have been reported on the surface of ZnO particles^45^: (i) the 1610–1630 cm^−1^ band is due to bending H-O-H vibration bands of chemisorbed water^62^, (ii) the 3000–3650 cm^−1^ band is due to the reversible dissociative absorption of hydrogen on Zn as well as O site. The peak at 758 cm^−1^ can be attributed to the C-O bond stretching. These results prove that the ZnO-NPs retained their structures even after being subjected to various stirring conditions.

### Thermal analysis of ZnO nanoparticles

**Figure 4:**
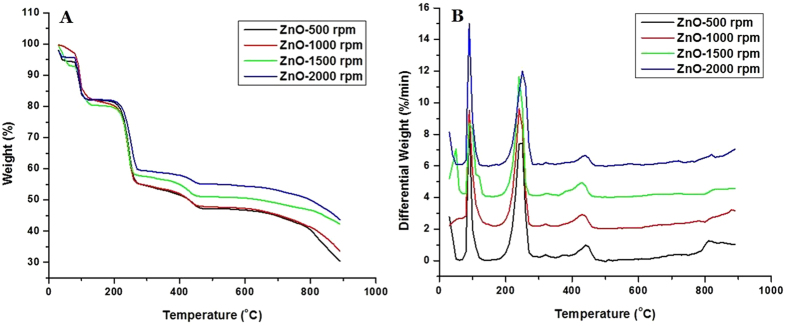
(**A**) TGA spectra of ZnO nanoparticles prepared at different stirring conditions. (**B)** DTA spectra of ZnO nanoparticles prepared at different stirring conditions.

The thermal properties of ZnO-NPs were determined by differential thermal analysis (DTA)and thermogravimetric analysis (TGA). Sample was heated in an aluminium pan upto 900 °C at the rate of 10 °C/min. The thermo-gravimetric analysis (TGA) curves (Fig. 4A) revealed the weight loss in two main temperatures ranges, (90 °C–120 °C) and (240 °C–280 °C). The weight loss around the temperature range (90 °C–120 °C) was ~ 17.08%, which is ascribed to the loss of hydroxyl group. In continuation, the weight loss around temperature range (240 °C–260 °C) was ~ 38.77%, which is accredited to the loss of acetate groups. It is seen that the residual weights of ZnO-500 rpm, ZnO-1000 rpm, ZnO-1500 rpm and ZnO-2000 rpm at 450 °C are 48.14, 48.30, 51.34 and 55.55%, respectively (Table 2). Thus, it can be said that the sample ZnO-2000 rpm is more thermally stable than the other samples.

DTA curves of ZnO-NPs were found to exhibit three exothermic peaks at 100 °C, 250 °C and 450 °C (Fig. 4B). The exothermic peaks at 100 °C correspond to decomposition stage between 90 °C and 120 °C while the exothermic peak at 250 °C corresponds to second decomposition stage (240 °C to 260 °C) in TGA (Fig. 4B). However, the exothermic peak at 450 ^o^C corresponds to third decomposition stage (420 °C to 470 °C) in TGA (Table 3). DSC analysis of ZnO-NPs prepared at different rpm was studied as a function of rate of weight loss (w/g) versus temperature (Supplementary Fig. 1). In case of ZnO-500 rpm, ZnO-1000 rpm and ZnO-1500 rpm decomposition at 100 °C and 250 °C was found with −13.95, −16.43 and −6.20 w/g weight loss respectively. However, in the case of ZnO-2000 rpm the decomposition was observed only at 100 °C with −3.624 w/g weight loss^63^.

### Growth mechanism of ZnO nano-thorns

The growth of ZnO nano-thorns follows similar pattern as that of ZnO nano-needles reported earlier^16^ and could be easily explained by the following reaction mechanism:


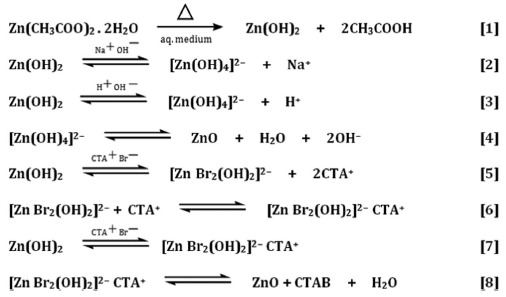


In a typical reaction, freshly synthesized Zn(CH_3_COO)_2_·2H_2_O solution was gradually added to previously prepared CTAB solution under different stirring conditions; this mixing produces zinc cation which further reacts with hydroxyl ion to form Zn(OH)_2_^16^. Under basic conditions, Zn(OH)_2_ is sparingly soluble and exists mainly in the form of a complex [Zn(OH)_4_]^2−^ and leads to the development of some active reaction pockets. In alkaline pH, the nucleation (formation of small growth units) step directs bidirectional growth^64^ on the positive <0 0 0 I> and negative <0 0 0 Ī> faces of the crystal due to dipole interaction, generally as a function of the growth rate (G), as follows:


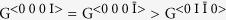


The formation of (Zn[OH]_4_)^2−^ intermediates thus directs the development of elongated (rod-shaped) ZnO-NPs arrays, bounded mainly by hexagonal prism <0 I Ī 0>.

**Figure 5:**
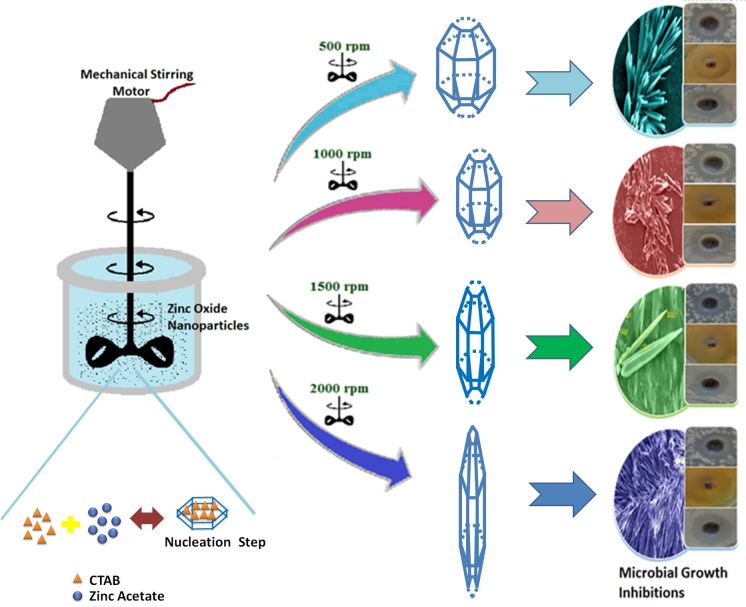
Schematic illustration portraying Sol-gel synthesis of thorn-like ZnO nanoparticles endorsing mechanical stirring effect.

The surfactant CTAB not only hastens the formation of the growth units, but also directs their two-sided growth^65^. Moreover, it also reduces the solution’s surface tension, which in turn decrease the energy needed for the formation of a new phase. The diverse types of developed complexes of (Zn[OH]_4_)^2−^ and CTAB get adsorbed on the ZnO surfaces due to electrostatic attractions, thus resulting in the elongation of c-axis (Fig. 5). This in turn actively extends primarily towards the <0 0 0 I> facet, thus leading to an increase in the number of spiny particles (possessing good aspect ratio)^39^,^66^,^67^,^68^. This anisotropic growth leads to the development of sharp thorn-like particles.

Variation in synthesis conditions like Zn^2+^ concentration and pH of the solution have been reported to influence the stability and relative growth rates of different crystal planes, thus affecting the morphology of ZnO nanoparticles^69^. In our experiment, the solutions were deliberately stirred at varying speeds in order to determine its effect on the growth of ZnO nanothorns. Mechanical stirring leads to the shear and better dispersion of nanoparticles. Higher agitation speed may disturb the metal ion nucleation more extensively which minimizes the particle agglomeration^70^, thus decreasing the size. It has been shown that change in stirring speed results in variable cluster size of nanoparticles^71^. Hence, high agitation rate may well disperse the particles being reduced, thus facilitating the interaction of individual particles with the surfactant, thereby reducing the particle diameter.

Some of the benefits of this technique include the simple and economical synthesis methodology, the moderate reaction temperature, use of conventional mechanical stirring conditions and the lack of need for inert atmosphere^72^. It also does not involve high temperature and high pressure for the initiation of the reaction. Moreover, the utilization of certain toxic substances (that are essential ingredients for some reaction processes) is not required.

### Potential role of ZnO-NPs as nano-antibiotics

The studies where ZnO-NPs have demonstrated its anti-pathogenic behavior are plentiful^16^,^38^,^63^,^73^,^74^,^75^,^76^,^77^,^78^. Its activity has been tested against a plethora of disease causing pathogens, like *Salmonella enteritidis*, *C. albicans*, *Listeria monocytogenes*, and *Pseudomonas aeruginosa*. Similarly, the ZnO nanoparticles have also presented good results in the protection of food and agriculture products against *B. subtilis, E. coli, Botrytis cinerea and Penicillium expansum.* Keeping this fact in mind, our in-house prepared ZnO-NPs were further evaluated for their anti-bacterial as well as anti-fungal efficacy using model microorganisms.

The antimicrobial activity was evaluated in terms of the zone of inhibition on NA plates. A significant reduction in the growth of microbial stains (*B. subtilis*, *E. coli*, and *C. albicans*) was observed at highest concentration (0.50 mg/mL) (Table 4). A maximum zone of inhibition (23 mm) was recorded for *B. subtilis* by ZnO-2000 rpm. Our preliminary data also suggests that the antimicrobial activity of ZnO-NPs depends upon the morphology and size of ZnO-NPs, which in turn depends on the stirring rate. ZnO-2000 rpm being smaller in size readily diffuses into the growth medium, allowing greater interaction between the NPs and the pathogen, hence exhibits better microbicidal property. Similar biological activity has been reported for ZnO-NPs synthesized from different approaches^53^. It can be also concluded that the Gram–positive stains are more susceptible than Gram-negative strain, which can be attributed to the presence of special cell wall structure containing different charged components. It has been suggested that some nanoparticles might interact with the thiol groups of crucial enzymatic machineries, hence leading to the loss of activity^53^. Even though this might happen, it would be hard to predict the exact location where these NPs strike the microbial cells.

**Figure 6:**
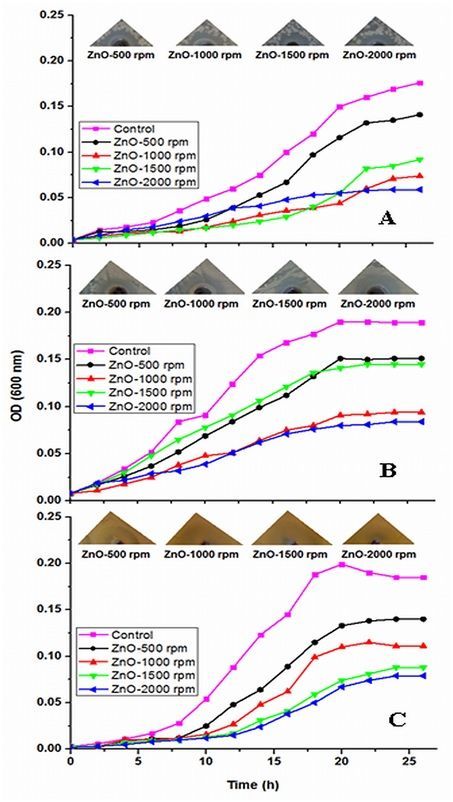
Microbial growth curve of (**A**) Bacillus subtilis, (**B**) Escherichia coli and (**C**) Candida albicans in the presence of ZnO nanoparticles (ZnO-NPs) prepared at different stirring conditions. The upper panel corresponds to the pictures of microbial growths in Petri plates.

The relative potency of the ZnO-NPs against microbes was also assessed by its MIC value (Supplementary Table 1), which was found to be 8 μg/ml in all the cases. Furthermore, noticeable difference in growth rate has been observed for all the microbes after 4 h of incubation with 0.5 mg/ml of ZnO-NPs (Fig. 6). The logarithmic growth phase of all the pathogens were found to be prolonged starting from 5 h to more than 20 h of incubation (Fig. 6). ZnO-NPs exhibited broad spectrum antibacterial activity against Gram-positive (*B. subtilis*) as well as Gram-negative (*E. coli*) strains. These findings are indicative of the fact that the inhibitory efficacy of ZnO-NPs is directly proportional to the stirring rate during the synthesis. Earlier studies have similarly demonstrated the role of size and morphology of ZnO-NPs on its antimicrobial activity^16^. Though *B. subtilis* is not as detrimental as other pathogenic bacteria, it serves as a model for performing the pre-clinical anti-bacterial efficacy studies, as the mechanism of action on the Gram positive bacteria remains the same. Similarly, the effect of ZnO-NPs on the viability of *C. albicans* was also tested. It can be seen that for ZnO-NPs prepared at 2000 rpm, the growth reduction was highest followed by ZnO-NPs synthesized at 1500 rpm, 1000 rpm and 500 rpm (Fig. 6). The results suggest that ZnO-NPs have a marked activity against *C. albicans*, with cytotoxic effect being dependent on the stirring rate. These findings indicate that ZnO-NPs can be applied superficially as a topical formulation against the pathogens.

In most of the dangerous microbes, the cell surface components such as proteins, polysaccharides and teichoic acids, offer protection against host defenses and environmental conditions^79^. Apparently, recent studies has revealed that certain long-chain polycations coated onto the surfaces can efficiently eradicate Gram-positive as well as Gram-negative bacteria on contact^80^,^81^. Researchers have proposed a number of modes of interaction between microorganisms and NPs. The antimicrobial mechanism of ZnO-NPs is primarily based on formation of reactive oxygen species like hydroxyl radicals, hydrogen peroxide, and superoxide anions, which can damage cell membrane and result in cell leakage and enhanced permeability^82^,^83^,^84^. Electrostatic binding of ZnO particles to bacterial surface kills the pathogens by disrupting the bacterial outer membrane^11^. Several studies have specified that ZnO-NP’s cell surface interactions affect permeability as well as morphology of cell membrane^63^,^76^,^85^. As a result, the introduction of ZnO-NPs inside pathogens provokes oxidative stress, ensuing in cell growth inhibition and, ultimately, cell death. However, the exact mechanism of the toxicity has yet to be ascertained.

## Conclusion

The present study encompasses the synthesis of thorn-like ZnO-NPs, prepared at different stirring conditions (viz. 500 rpm, 1000 rpm, 1500 rpm and 2000 rpm) and the effect of mechanical stirring on the fabrication of ZnO-NPs. Results show that the speed of agitation in the synthesis protocol is a critical value in determining the size of the particles. It can be concluded that the rotation speed has a consistent impact on the aspect ratio of ZnO-NPs. The anisotropic growth of ZnO-NPs is promoted by stirring, possibly through the induction of internal shear force. Thus, the morphology and size of ZnO-NPs can be tuned by simply adjusting the stirring speed. The in-house synthesized nanoparticles displayed thorn like morphology with wurtzite crystal structure and displayed high thermal stability. Significant antimicrobial activity reveals that the ZnO nano-antibiotics may be used safely in anti-fungal and anti-bacterial therapeutic ointments, creams, etc. However, the efficacy of these nanostructures is needed to be evaluated under *in-vivo* conditions in experimental animal models before introducing them into clinical settings.

## Additional Information

**How to cite this article**: Khan, M. F. *et al*. Sol-gel synthesis of thorn-like ZnO nanoparticles endorsing mechanical stirring effect and their antimicrobial activities: Potential role as nano-antibiotics. *Sci. Rep.* **6**, 27689; doi: 10.1038/srep27689 (2016).

## Supplementary Material

Supplementary Information

## Figures and Tables

**Table 1:** The effect of various reaction conditions at different stirring conditions on the synthesis and characteristics of thorn-like ZnO nanoparticles.

**Sample**	**Nano-diameters (nm)**	**Aspect ratio**
ZnO-500 rpm	~25 nm	~8.6
ZnO-1000 rpm	~20 nm	~9
ZnO-1500 rpm	~7 nm	~13
ZnO-2000 rpm	~3 nm	~18

**Table 2:** Thermogravimetric analysis (TGA) of thorn-like ZnO nanoparticles at different stirring conditions.

Sample/Temperature	**Residual weight (%)**		
	**100 °C**	**250 °C**	**450 °C**
ZnO-500 rpm	83.92	62.23	48.14
ZnO-1000 rpm	86.19	60.03	48.30
ZnO-1500 rpm	84.51	61.03	51.34
ZnO-2000 rpm	84.30	69.08	55.55

**Table 3:** Differential thermal analysis (DTA) of thorn-like ZnO nanoparticles at different stirring conditions.

Sample/Temperature	**Differential weight (%/min)**		
	**100 °C**	**250 °C**	**450 °C**
ZnO-500 rpm	1.1974	7.441	0.8811
ZnO-1000 rpm	3.619	6.397	0.4557
ZnO-1500 rpm	4.327	5.719	0.2834
ZnO-2000 rpm	2.891	6.001	0.4863

**Table 4:** Antimicrobial activity of thorn-like ZnO nanoparticles at different stirring conditions.

**Samples**	Concentration(mg/ml)	**Zone of inhibition (mm)**		
		***B. subtilis*****(MTCC 121)**	***E. coli*****(ATCC 25922)**	***C. albicans***
ZnO-500 rpm	0.50	16 ± 1.7	15 ± 2.3	20 ± 1.5
ZnO-1000 rpm	0.50	20 ± 2.4	17 ± 1	22 ± 1.8
ZnO-1500 rpm	0.50	22 ± 1.5	18 ± 2.6	23 ± 3
ZnO-2000 rpm	0.50	23 ± 2.4	19 ± 1.3	25 ± 2
ZnO Powder	0.50	9 ± 0.8	7 ± 1	3 ± 0.5
Control	–	0	0	0
Doxycycline	0.03 mg/disc	30 ± 2.5	21 ± 1	–
Nystatin	0.03 mg/disc	–	–	20 ± 1.6

The data represents mean values of three independent experiments ± SD.
