# Supplementary material for: Sol-gel synthesis of thorn-like ZnO nanoparticles endorsing mechanical stirring effect and their antimicrobial activities: Potential role as nano-antibiotics
Source: Sci Rep. 2016 Jun 28;6:27689. doi: 10.1038/srep27689 (PMC4923881; doi:10.1038/srep27689)
Supplement: Supplementary Information [file srep27689-s1.doc]

**Sol-gel synthesis of thorn-like ZnO nanoparticles endorsing mechanical stirring effect and their antimicrobial activities: Potential role as nano-antibiotics**

Mohd Farhan Khan^1,2^, Akhter H. Ansari^1^, M. Hameedullah^1^, Ejaz Ahmad^3^, Fohad Mabood Husain^4,5^, Qamar Zia^6,7^, Umair Baig^8^, Mohd Rehan Zaheer^2^, Mohammad Mezbaul Alam^9^, Abu Mustafa Khan^10^, Zeid A. AlOthman^9^, Iqbal Ahmad^4^, Mohammad Owais^6^, Ghulam Md Ashraf^11^, Gjumrakch Aliev^12,13,14,*^

*^1^Nano Solver Lab, Department of Mechanical Engineering, Z. H. College of Engineering & Technology, Aligarh Muslim University, Aligarh-202002, India; ^2^Faculty of Science, Gagan College of Management & Technology (GCMT), Aligarh-202002, India; ^3^Department of Biological Sciences, University of Toledo, Toledo, OH43606, USA; ^4^Department of Agricultural Microbiology, Aligarh Muslim University, Aligarh-202002, India; ^5^Department of Food Science and Nutrition, College of Food and Agriculture, King Saud University, Riyadh-11451, Kingdom of Saudi Arabia; ^6^Interdisciplinary Biotechnology Unit, Aligarh Muslim University, Aligarh-202002, India; ^7^Department of Biotechnology, Gagan College of Management and Technology (GCMT), Aligarh, India; ^8^Center of Excellence for Scientific Research Collaboration with MIT, King Fahd University of Petroleum and Minerals, Dhahran 31261, Saudi Arabia; ^9^Advanced Materials Research Chair, Chemistry Department, College of Sciences, Building 5, King Saud University, Riyadh-11451, Kingdom of Saudi Arabia; ^10^Department of Chemistry, Aligarh Muslim University, Aligarh-202002, India; ^11^King Fahd Medical Research Center, King Abdulaziz University, Jeddah, Saudi Arabia; ^12^GALLY International Biomedical Research Consulting LLC., 7733 Louis Pasteur Drive, #330, San Antonio, TX, 78229, USA; ^13^School of Health Science and Healthcare Administration, University of Atlanta, E. Johns Crossing, #175, Johns Creek, GA, 30097, USA; ^14^Institute of Physiologically Active Compounds, Russian Academy of Sciences, Chernogolovka, 142432, Russia*

***Running Heads:*** *Nano-antibiotics as antimicrobial agents*

* Corresponding author

GA: Tel. +1(440) 263-7461; E-mail: [aliev03@gmail.com](mailto:aliev03@gmail.com), [GAliev@uofa.edu](mailto:GAliev@uofa.edu)

**Supplementary information**

*Antimicrobial activity of ZnO nanoparticles*

The antibacterial activity of ZnO-NPs was performed using the filter paper disc method^1^ as standardized in our lab^2^. All cultures were routinely maintained on nutrient agar (NA) and incubated at 37°C overnight. The culture was centrifuged at 1,000 rpm, and pellets were resuspended and diluted in sterile normal saline solution to obtain the viable count of 10^5^ cfu/mL. Diluted bacterial culture suspension (0.1 mL) was spread uniformly, with the help of a spreader, on NA plates. Sterile 8 mm discs (HiMedia Laboratories Pvt Ltd, Mumbai, India) were impregnated with the ZnO-NPs. The plates were then incubated at 37°C for 24 hours. The antifungal activity of ZnO-NPs was also performed using the same methods described previously, but with slight differences.

All cultures were routinely maintained on sabouraud dextrose agar (SDA) and incubated at 28°C. Further, the inoculums of non-sporing fungi, *C. albicians* were performed by growing the culture in Sabouraud dextrose broth at 37°C overnight. A total of 0.1 mL of diluted fungal culture suspension was spread uniformly, with the help of a spreader, on SDA plates. C. albicans plates were incubated at 37°C for 18–48 hours. An antibiotic disc (0.03 mg/disc; HiMedia Laboratories Pvt Ltd), doxycycline (for bacteria) and nystatin (for fungi) were used as standard. Media with ZnO powder of same concentration was set up as the control. The diameters of the resulting inhibition zones (in mm) of microbial growth were measured for the determination of antibacterial and antifungal activities^3^.

For growth inhibition kinetics, the bacteria were first grown on solid nutrient agar medium. Then, fresh colonies were picked from the agar plates and inoculated into 100 ml of nutrient broth (NB) medium. Growth was monitored at every 2 h under UV-visible spectrophotometer (Electronics Corporation of India Limited, UV-2550) till 26 h. Similarly, antifungal tests were performed by measuring the growth curve of *C. albicans* incubated in Yeast, peptone, dextrose (YPD) medium containing different ZnO-NPs and growth was read every 2 h. The experiments were repeated thrice and results are expressed as mean±SD.

The broth microdilution method was used as the refer­ence method, as described by the Clinical and Laboratory Standards Institute (document M27-A24)^4^ (**CLSI 2002**). The inoculum was prepared from Sabouraud dextrose agar subcultures incubated at 35°C for 24 hours and the resulting suspension was adjusted spectrophotometrically to a density equivalent to a 0.5 McFarland standard at 530 nm (1.5×10^6^ colony forming units [CFU]/mL). A working suspension was made by 1:100 dilution of the suspension in water with L-glutamine and without bicarbonate (Sigma-Aldrich), buffered to pH 7.0 with 0.165 M morpholinepropanesulfonic acid (Sigma-Aldrich). Next, 100 μL of the diluted inoculum was added to each well. The final inoculum size was (0.5–2.5) 103 CFU/mL. The broth dilution tests were incubated at 35°C, and minimum inhibitory concentrations (MICs) were determined after 48 hours of incubation by observation of the presence or absence of visible growth[^5^](#_ENREF_3).

The minimal inhibitory concentration (MIC) of the ZnO-NPs was evaluated following the broth macro-dilution method M26-A described by the Clinical and Laboratory Standards Institute (CLSI). Briefly, a bacterial suspension in a mid-exponential phase was inoculated into 10 mL of Mueller-Hinton Broth **(**MHB) starting with an initial inoculum size of approximately 5 × 10^5^ CFU/mL. Different concentrations of the ZnO-NPs were added and the tubes were incubated at 37 °C for 20 h. The MIC is the lowest concentration of antimicrobial agent that completely inhibits growth of the organism in the tubes or microdilution wells as detected by the unaided eye^6^.

*References*

1. Bauer AW, Kirby WM, Sherris JC, Turck M. Antibiotic susceptibility testing by a standardized single disk method. Am J Clin Pathol. 1966;45(4):493–496
2. Musthafa TNM, Siddiqui ZN, Husain FM, Ahmad I. Microwave-assisted solvent-free synthesis of biologically active novel heterocycles from 3-formylchromones. Med Chem Res. 2011;20(9):1473–1481.
3. Zia Q, Khan AA, Zubair S, Owais M. Self-assembled amphotericin B loaded poly-glutamic acid nanoparticles: Preparation, characterization and in vitro potential against Candida albicans. Int J Nanomed, 2015;10:1769–1790.
4. Khan, M. F., Hameedullah, M., Ansari, A. H., Ahmad, E., Lohani, M. B., Khan, R.H., Alam, M.M., Khan, W., Husain, F.M. & Ahmad, I. Flower-shaped ZnO nanoparticles synthesized by a novel approach at near-room temperatures with antibacterial and antifungal properties. Int. J. Nanomed. 9, 853-864 (2014).
5. Clinical and Laboratory Standards Institute (CLSI) CdM-A. Reference method for broth dilution antifungal susceptibility testing of yeasts; approved standard-method-Second Edition (ISBN 1-56238-469-4). Wayne, PA: CLSI. 2002;Vol. 22(No. 15).
6. National Committee for Clinical Laboratory Standards. Methods for dilution antimicrobial susceptibility tests for bacteria that grow aerobically; *—Fifth Edition: Approved Standard M7-A5* (PA): NCCLS; 2000

**Supplementary table 1:** MIC values of ZnO-nanoparticles prepared under different stirring conditions

| **Samples** | **Concentration (µg/ml)** | **MIC ( µg/ml)** | | |
| --- | --- | --- | --- | --- |
|  |  | ***B. subtilis***  **(MTCC 121)** | ***E. coli***  **(ATCC 25922)** | ***C. albicans*** |
| **ZnO-500 rpm** | 500 | 8 | 8 | 8 |
| **ZnO-1000 rpm** | 500 | 8 | 8 | 8 |
| **ZnO-1500 rpm** | 500 | 8 | 8 | 8 |
| **ZnO-2000 rpm** | 500 | 8 | 8 | 8 |
|  |  |  |  |  |


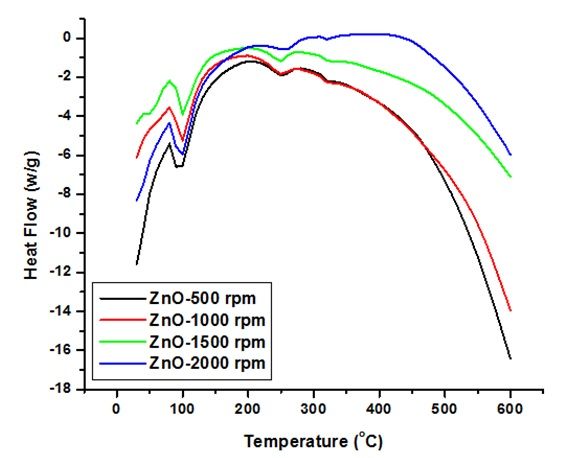


Figure. DTA spectra of ZnO nanoparticles prepared at different stirring conditions.
